# Role of Th17 cells and IL-17, IL-23 cytokines in pathogenesis of autoimmune thyroid disease in children

**DOI:** 10.1186/1756-6614-6-S2-A8

**Published:** 2013-04-05

**Authors:** Artur Bossowski, M Moniuszko, M Dabrowska, M Rusak, M Jeznach, A Bodzenta-Łukaszyk, A Bossowska

**Affiliations:** 1 Department of Pediatrics Endocrinology, Diabetology with Cardiology Division, Medical University of Białystok, Poland; 2 Department of Allergology and Internal Medicine, Medical University of Białystok, Poland; 3 Department of Hematological Diagnostics, Medical University of Białystok, Poland; 4 Division of Cardiology, Internal Affairs Ministry Hospital in Białystok, Poland

## Introduction

Up till now, altered balance of Th1 and Th2 immune cells has been postulated to play an important role in the pathogenesis of autoimmune thyroid diseases (AITD). However, recent studies on thyroid diseases suggest a new role for Th17 (T helper 17) cells that have been classified as a new lineage, distinct from Th1, Th2 and Treg cells. Despite wide interest, the role of Th17 cells in the pathogenesis of inflammatory and autoimmune diseases is still being debated. Th17 cells are involved in immune responses against extracellular pathogens and have the ability to secrete cytokines: IL-17, IL-17F, IL-21 and IL-23. Th17 cells can be characterized by several surface markers, i.e. CCR6 (CD196), IL-23R, IL-12Rbeta2 and CD161.

## Aim of the study

Was to estimate the proportions of circulating CD4+CD161+CD196+ and CD4+IL-17+ Th17 cells and serum concentrations of IL-17 & IL-23 in patients with Graves' disease (GD, n=22, mean age ± SEM 14.3 ± 4 years), Hashimoto's thyroiditis (HT, n=37, mean age ± SEM 15 ± 2 yrs) and in healthy controls (C, n=25, mean age ± SEM 15.2 ± 2 yrs).

## Material and methods

Polychromatic flow cytometry and several fluorochrome-conjugated monoclonal antibodies were applied to delineate Th17 cells with either CD4+CD161+CD196+ or CD4+IL-17+ phenotype using apparatus FACSCalibur (BD Biosciences). The expression of IL-17 and IL-23 were analyzed by Bio-Tek ELx800 ELISA reader. Thyroid anti-TSH receptor immunoglobulins (TRAK), anti-thyroperoxidase (anti-TPO) and anti-thyroglobulin (anti-TG) antibodies were measured in all the samples using electrochemiluminescence "ECLIA" with Modular Analytics E170 analyzer (Roche Diagnostics, Poland).

## Results

In untreated HT children we observed an increased percentage of CD4+CD161+CD196+ (7.1±3.5 vs. 3.7±1.8; p <0.04) and CD4+IL-17+ (3.7±2.7 vs. 1.4±0.4; p <0.01) Th17 lymphocytes in comparison to the healthy controls. In GD children we did not reveal such abnormalities in the population of these cells. In cases with HT, a positive correlation between the percentage of CD4+IL-17+ and CD4+CD161+CD196+ T cells and serum level of anti-TPO antibodies (r=0.48; p <0.025; r=0.65; p <0.01; respectively) was detected. In untreated patients with AITD we observed an increased levels of IL-23 in comparison to control group (GD:156.29±118.22 vs. 69.04±38.23, p=0.004, HT:135.04±140.19 vs. 69.04±38.23, p=0.046). Methimazole treatment in GD led to decrease of these cytokine levels in a period of 6-12 months. However, during 6-24 months of L-thyroxine therapy in HT there wasn’t any reduction of IL-23 concentration compared with HC. IL-17 was elevated only in HT patients in comparison to the controls (17.17±10.49 vs. 11.38±2.99, p=0.021), which normalized during therapy.

## Conclusions

We conclude that the increased percentage of Th17 cells and elevated level of IL-17 and IL-23 cytokines in children with HT can suggest their role in initiation and development of immune and inflammatory processes in this endocrinopathy.

